# High-throughput profiling of the IgG and IgA response to the *Treponema pallidum* subsp. *pallidum* proteome in syphilis patients

**DOI:** 10.1128/mbio.00820-26

**Published:** 2026-06-09

**Authors:** Lorenzo Giacani, Linda H. Xu, Amit Oberai, Jozelyn V. Pablo, Christopher Hung, Andy A. Teng, Adam D. Shandling, Kelika A. Konda, Silver K. Vargas, Michael Reyes Diaz, Carlos F. Caceres, Jeffrey D. Klausner, Joseph J. Campo

**Affiliations:** 1 Department of Medicine, Division of Allergy and Infectious Diseases, University of Washington 7284, Seattle, Washington, USA; 2 Department of Global Health, University of Washington 7284, Seattle, Washington, USA; 3 Antigen Discovery, Inc., Irvine, California, USA; 4 Center for Interdisciplinary Studies in Sexuality, AIDS and Society, Universidad Peruana Cayetano Heredia, Av. Honorio Delgado, Lima, Peru; 5 Department of Population and Public Health Sciences, Keck School of Medicine, University of Southern California 5116, Los Angeles, California, USA; Cornell University, Ithaca, New York, USA

**Keywords:** *Treponema pallidum*, syphilis, protein array, serodiagnosis, staging, treatment response

## Abstract

Syphilis continues to be a significant global health concern. To define the extent of the IgG and IgA antibody reactome in patients with syphilis and identify reactive antigens and reactivity patterns that could facilitate early diagnosis and possibly serve as biomarkers for treatment response and staging, we developed a pan-proteomic microarray carrying 98.8% (1,009 antigens) of the annotated polypeptides of two *Treponema pallidum* subsp*. pallidum* strains (Nichols and SS14). In this study, we probed the array with 217 sera collected pre- and post-treatment from 122 syphilis patients to detect IgG and IgA antibodies. Although a minimal IgA response to *T. pallidum* antigens were detected in these sera, with only 21 reactive targets on the array, corresponding to 20 *T. pallidum* antigens, significant IgG reactivity was detected to a total of 104 array protein spots, corresponding to 101 *T. pallidum* antigens, and roughly encompassing 10% of the pathogen’s proteome. No targets were differentially recognized in pre-treatment sera when factoring in covariates such as syphilis stage, syphilis history, and human immunodeficiency virus status, but a significant decrease in reactivity to 82 antigens was detected in the sera collected post-treatment compared to sera collected at diagnosis. This work helped define the extent of the IgG and IgA antibody response in syphilis patients and identified antigens that could be further evaluated to improve treponemal serological tests. Antigens inducing a detectable IgA response in adults, albeit very few, could be further investigated as diagnostic markers.

**IMPORTANCE:**

This investigation improved our understanding of the IgG and IgA humoral response to virtually all proteins of *Treponema pallidum* subsp. *pallidum* (*T. pallidum*) during natural infection in patients with active and treated syphilis. Using samples collected pre- and post-treatment from individuals presenting with syphilis at different stages, human immunodeficiency virus status, and history of recurrent infection, we identified antigens that could be further investigated to improve early syphilis and congenital syphilis diagnosis and serve as possible biomarkers to monitor treatment response.

## INTRODUCTION

Syphilis is a multistage chronic sexually transmitted infection (STI) still representing a significant burden for global health despite being curable. Undiagnosed syphilis can progress to affect a patient’s cardiovascular and central nervous systems, leading to severe clinical manifestations and, occasionally, death (1). Furthermore, during pregnancy, the syphilis agent, *Treponema pallidum* subsp. *pallidum* (*T. pallidum*), can cross the placenta and infect the fetus, potentially leading to fetal demise (2).

The global syphilis burden has been estimated to be up to 70 million cases (3–7), while according to the World Health Organization (WHO), approximately 8 million new syphilis cases occurred in adults aged 15–49 years in 2022 (8). Although most cases occur in low- and middle-income countries (LMICs), syphilis rates have increased over the last few decades in high-income nations as well (9–14). In the United States, where syphilis has been on the rise since 2000, the rate of early (primary and secondary) syphilis went from 2.1 cases per 100,000 population in 2000 to 15.8 cases per 100,000 population in 2023 (15), and the increasing syphilis rates in women of reproductive age have led to a dramatic increase in the incidence of congenital syphilis (CS) between 2000 (529 cases of CS; 13.4 per 100,000 live-born infants) and 2023 (3,882 cases of CS; 105.8 per 100,000 live-born infants) as reported by the Centers for Disease Control and Prevention. On a global scale, congenital syphilis continues to impact pregnancy outcomes significantly (16). In 2016, for example, ~200,000 stillbirths and perinatal deaths were attributed to congenital syphilis worldwide, particularly in LMICs (2, 17, 18), while recent estimates from the WHO indicate 700,000 cases of CS in 2022 (8).

The global burden of syphilis and its epidemiological trends warrant pursuing research endeavors that could translate into new ways to control syphilis. Such efforts include the development of an effective vaccine to prevent or attenuate the overall clinical picture associated with the infection, drug repurposing to widen the therapeutic options to treat syphilis and counteract the shortages of benzathine penicillin G (BPG) (19, 20), and improving current molecular and serological tests to achieve early diagnosis, monitor treatment efficacy, and facilitate the diagnosis of active syphilis in asymptomatic newborns from mothers exposed to syphilis during pregnancy.

Because (i) a primary syphilitic chancre is generally not painful and can be missed during physical exams, especially if concealed within a patient’s cervix, oropharynx, or rectum; (ii) the protean symptoms of the secondary stage can lead to misdiagnosis; and (iii) patients with latent syphilis are not symptomatic despite being actively infected, syphilis is usually diagnosed using a combination of serological assays (21). These are divided into lipoidal and treponemal tests. Lipoidal tests, such as the rapid plasma reagin (RPR) test, are flocculation assays that measure antibodies elicited by lipoidal material released from both damaged host cells and the bacterial envelope. However, because up to 2 weeks from the appearance of the primary lesion might be necessary for a lipoidal test to become positive, about 25%–30% of early cases may be missed (22–24). In the absence of treatment, lipoidal antibody titers generally peak 1–2 years after infection and remain positive even in late disease (25, 26). After treatment, titers decline and generally become nonreactive within 6 months in immunocompetent patients, although seroreversion might require up to 2 years in some cases (27). Up to ~20% of syphilis patients show persistent low-titer lipoidal antibodies (a condition known as serofast state) even after therapy appropriate for their disease stage (28). The achievement of serological cure post-treatment is defined as a 4-fold decrease in lipoidal antibody titers, while titer increase post-treatment is associated with reinfection or relapse (25, 26, 29).

**Fig 1:**
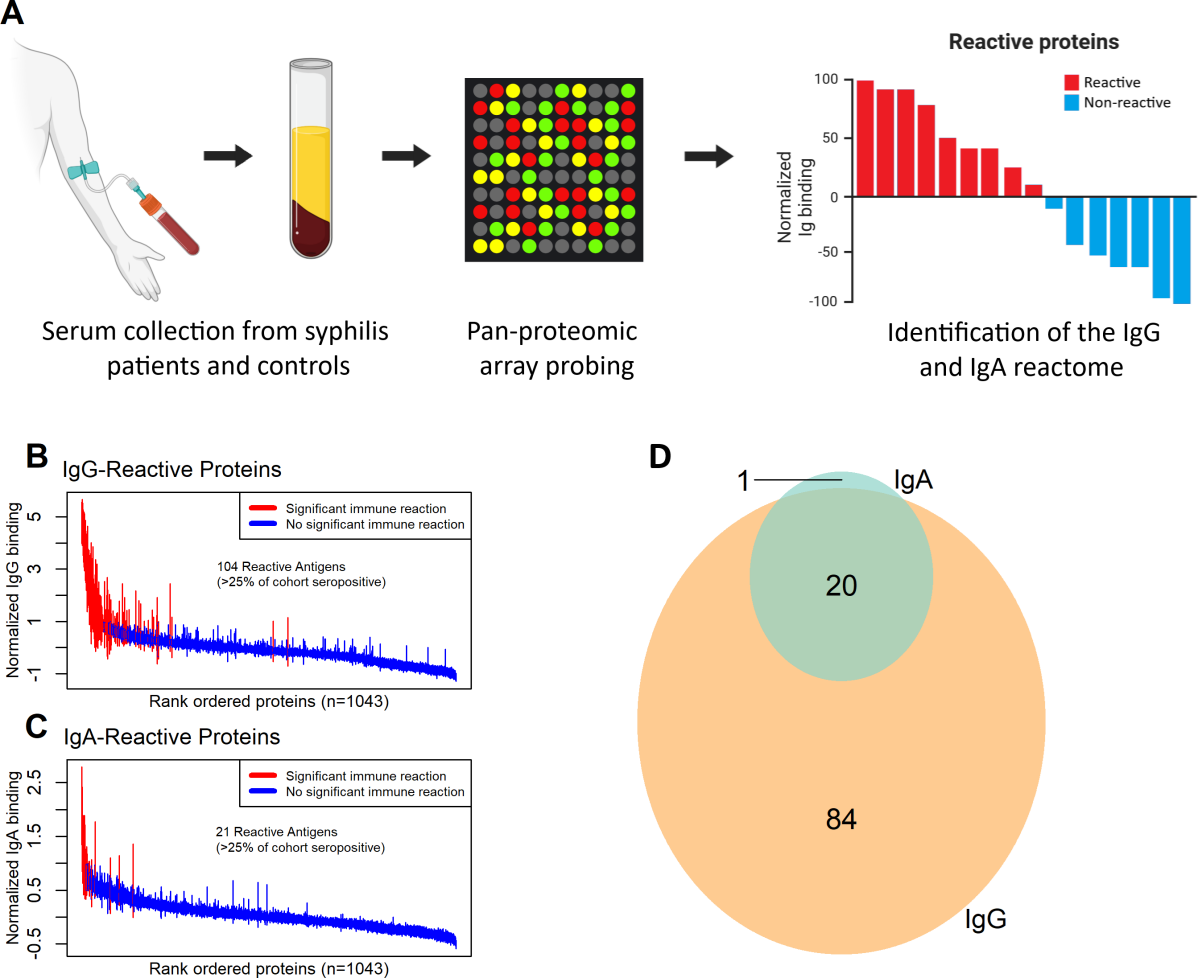
Overall IgG and IgA reactivity in sera from syphilis patients. (**A**) Experimental design. Serum samples from negative controls and patients with syphilis at different stages, human immunodeficiency virus (HIV) status, and history of syphilis were used to probe the pan proteomic array carrying 1,009 antigens representing the proteome of two *T. pallidum* strains (Nichols and SS14) to identify the IgG and IgA reactome in infected individuals. (**B and C**) Interquartile range plots showing the maximal normalized IgG- (**B**) and IgA-binding (**C**) signal for each patient for each target on the array. Each bar represents a target on the array, and targets are ordered by the mean signal generated by testing each serum sample. Red bars represent printed proteins on the array with a significant seropositive response (i.e., above background) in at least 25% of the sera analyzed. Normalized IgG binding (Y axis) is expressed as the log2 signal-to-noise ratio, where a value of 0 represents a specific antibody SI equal to the background, 1.0 represents twice the background, 2.0 represents 4-fold over the background, and so forth. (**D**) Overlap in IgG and IgA antibody responses in patient sera. The Venn diagram shows IgA-reactive antigens in the green circle and IgG-reactive antigens in the yellow circle. All IgG-reactive antigens also elicited a significant IgA response except for Tp0445-Pfp1, a putative redox sensor, for which there was no matching IgG reactivity.

Treponemal tests such as the *T. pallidum* particle agglutination and *T. pallidum* hemagglutination tests use whole *T. pallidum* cell extracts to detect antibodies to virtually the entire pathogen proteome (29). In recent years, treponemal tests using only a subset of immunodominant *T. pallidum* antigens such as the Tp0574 (TpN47), Tp0435 (TpN17), and Tp0171 (TpN15) lipoproteins (used individually or in chimeric concatemers) in enzyme and chemiluminescence immunoassays (EIA and CIA, respectively) have become widespread in laboratories performing large-scale serological testing, as these assays are automated and results do not require subjective interpretation by the operator (30). Point-of-care treponemal tests are also generally based on the same recombinant antigens. Because treponemal antibodies persist typically for the patient’s life, the available treponemal tests cannot discriminate between an active and a previously treated infection. Treponemal tests generally become positive between 6 and 14 days after chancre appearance and, therefore, may detect early syphilis cases undetected by lipoidal tests (29). Given the above limitations, and because past studies have supported the importance of evaluating additional *T. pallidum*-specific antigens to assess their diagnostic value compared to currently used targets (31, 32), we have developed an antigen array encompassing the annotated proteomes of the Nichols and SS14 *T. pallidum* isolates (33) and used to identify novel IgG- and IgA-immunoreactive antigens that could help discriminate active versus successfully treated infections or facilitate disease staging. A schematic depiction of this work is presented in the Graphical Abstract (Fig. 1A).

## RESULTS

### Overall sample analysis

Here, 217 pre-and post-therapy serum samples from 122 patients (Table 1) with syphilis at different stages, with and without a history of infection, and with different HIV statuses were collected and analyzed.

**TABLE 1:** Characteristics of the participants with syphilis enrolled in this study, Lima, Peru, 2019–2021

Characteristic	*n*	%
Age, yrs		
18–25	30	25
26–30	34	28
31–35	25	20
>35	33	27
Sexual identity		
Cis man^*a*^	102	84
Trans woman	16	13
Cis woman	4	3
Sex worker		
No	98	80
Yes	24	20
HIV status		
Infected	46	38
Uninfected	76	62
History of prior syphilis		
Previous TP infection	60	49
No previous TP infection	34	28
Unknown history	28	23
Syphilis clinical stage		
Primary	41	34
Secondary	9	7
Early latent	57	47
Late latent/unknown duration	13	11
Suspected tertiary	2	2

^*a*^ Only 3 were not men who had sex with men.

The analysis of the syphilis patient sera used in this study showed that during infection, 104 distinct targets on the array elicited an IgG response significantly above background (Fig. 1B), corresponding to roughly 10% of the annotated Nichols and SS14 proteomes. These antigens constitute the IgG reactome in sera collected from patients across four reliably assigned stages of infection (primary “I,” secondary “II,” early and late latent, and latent syphilis of unknown duration “EL/LL/U”; Table 1) and collected before treatment administration and at the 3- and 6-month follow-up visits post-treatment. The 104 distinct IgG-reactive targets correspond to 101 *T. pallidum* proteins, as the hypothetical protein Tp0855 was expressed in two distinct fragments of equal length representing the NH_2_- and the COOH- termini of the protein, respectively, and two pairs of proteins were isoforms of the same antigen. A complete list of the IgG-reactive antigens is reported in Table S1 in white-background cells organized hierarchically by seroprevalence in pre-treatment sera collected at the time of diagnosis (column H; Seropositivity, visit 0), while non-reactive antigens per our selection criteria are in gray-background cells. Most (97%) of the IgG-recognized antigens were members of the core *T. pallidum* proteome (i.e., proteins with identical amino acid sequence in both the Nichols and SS14 strains), except for Tp0479 (annotated as hypothetical protein), for which only the SS14 variant was significantly recognized, while the cognate Tp0479 antigen from Nichols did not meet our positivity criteria (Table S1). Both the Nichols and SS14 variants of the Tp0136 (putative fibronectin-binding adhesin) (34) and the Tp0131-TprD/TprD_2_ outer membrane proteins (OMPs) were significantly recognized by the patient sera (Table S1), likely because of the elevated amino acid sequence identity existing between the strain-specific variants of these antigens (Sequences in Table S1).

The analysis of the IgA reactome yielded 21 significantly recognized targets (Fig. 1C), corresponding to ~0.02% of the pathogen’s proteome and to 20 *T. pallidum* distinct proteins, given that two reactive targets on the array were fragments of the Tp0990 hypothetical protein. The complete list of the IgA-reactive antigens is reported in Table S2 in white-background cells organized hierarchically by seroprevalence at the time of diagnosis (column H; Seropositivity, visit 0), while non-reactive antigens are in gray-background cells. All IgA-reactive antigens were members of the *T. pallidum* core proteome. Table S1 (IgG reactivity) and Table S2 (IgA reactivity) also report seroprevalence at each follow-up visit, as well as the mean and median reactivity detected for each antigen at each time point. Table S3.1 and Table S3.2 report the reactivity for each target on the array in each individual serum sample analyzed for IgG and IgA, respectively.

Approximately 20% of the IgG-reactive antigens elicited an IgA response, while an IgA-positive signal was nearly always matched by a significant IgG reactivity for the same target, except for the putative sensor of oxidative stress Tp0445-Pfp1, for which there was no significant IgG signal (Fig. 1D). The same analysis performed on all control sera from patients without syphilis and with periodontal disease showed relatively mild but significant IgG reactivity to a total of 21 antigens (Fig. S1 and Table S4). More precisely, most IgG-positive targets (*n* = 20) were recognized by at least 25% of the non-syphilis sera (Fig. S1A), while only three antigens (Tp0567, Tp0868, and a fragment of the Tp0990 protein) were recognized by at least 25% of sera from patients with periodontal disease (Fig. S1B). Only one antigen (Tp0445) was IgA-positive and recognized by at least 25% of both non-syphilis and periodontal disease sera (Fig. S1C and S1D), and Table S4 provides intensity data for IgG- and IgA-reactive antigens for all control sera.

### Analysis of pre-treatment sera

**Fig 2:**
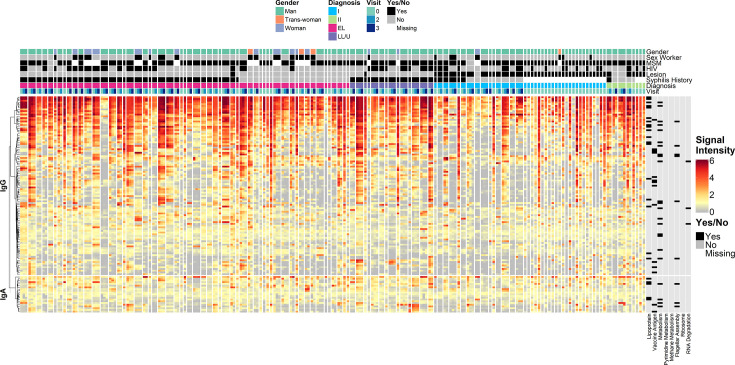
IgG and IgA reactivity profiles. The heat map shows the IgG and IgA binding SI for 104 IgG-reactive and 21 IgA-reactive antigens. Patient characteristics (gender, stage, visit number, HIV status, presence of lesion, etc.) are reported in the color-coded top rows (legend on top) unless data are missing (white gaps). Protein features identified by random forest and positively or negatively associated with reactivity to IgG or IgA are shown in black and gray columns to the right of the heat map (legend on right). Columns represent the serum samples ordered by subject and clinical visit. Rows represent *T. pallidum* proteins sorted by hierarchical clustering based on reactivity. Signal intensity is expressed as the log2 signal-to-noise ratio, where a value of 0 represents a specific antibody SI equal to the background, 1.0 represents twice the background, 2.0 represents 4-fold over the background, and so forth (legend on right). Legend: I: primary syphilis; II: secondary syphilis; EL: early latent syphilis; LL: late latent syphilis; U: latent syphilis of unknown duration. Visit 0: pre-treatment (diagnosis, enrollment, and treatment administration); Visit 2: 3-month follow-up post-treatment; Visit 3: 6-month follow-up post-treatment.

Protein feature selection associated with antigen reactivity using random forest algorithm identified lipoproteins (18/104 reactive proteins vs. 9/939 non-reactive proteins), vaccine candidates (12/104 vs. 14/939), flagellar assembly proteins (6/98 vs. 28/939), and RNA degradation proteins (3/104 vs. 6/939) as significantly represented in reactive proteins in 100 partitioned models. In contrast, metabolism-associated proteins (19/104 vs. 327/939), including pyrimidine metabolism (0/104 vs. 15/939) and methane metabolism (0/104 vs. 7/939), and ribosomal proteins (0/104 vs. 51/939) were significantly represented in non-reactive proteins (Fig. 2, Fig. S2, and Table S5). For IgG-reactive targets, Tp0768-TmpA, Tp0319-TmpC, Tp0435-TpN17, and Tp0574-TpN47 were among the most strongly recognized lipoproteins, while significantly reactive flagellar proteins included Tp0567-FlbB and Tp0727-FlgE. Among antigens encoding predicted OMPs/vaccine candidates, Tp0326-BamA, Tp0621-TprJ, Tp0117-TprC, and Tp0131-TprD were significantly reactive (Table S1). Similarly, IgA targets included lipoproteins such as Tp0768-TmpA, Tp0435-TpN17, Tp0319-TmpC, and Tp0433-Arp, flagellar proteins (Tp0727-FlgE, Tp0567-FlbB, Tp0398-FliE, Tp0663-FlaA, and Tp0872-FliD), OMPs (Tp0326-BamA and Tp0621-TprJ), and hypothetical proteins (Table S2).

To identify *T. pallidum* antigens that could possibly improve the sensitivity of treponemal tests and facilitate early syphilis diagnosis, the analysis of the IgG reactome was then limited to proteins that elicited a significant response in ≥85% of the sera collected pre-treatment and an overall SI ≥ 3. Such analysis yielded a total of 18 targets (Table 2). When analyzed by syphilis stage, three of these antigens, Tp0768-TmpA, Tp0567-FlBb, and Tp0319-TmpC, were reactive in >97% of both pre-treatment and primary sera. Four additional antigens, including the Tp0574-TpN47, Tp0259-LysM, Tp0684-MglB, and the hypothetical protein Tp0772, were reactive in fewer patients at collection time pre-treatment (96.7%–95.9%), albeit still >90% of the primary syphilis sera (94.7%–92.1%; Table 2). For comparison, the 17 KDa lipoprotein (Tp0435-TpN17), a widely used diagnostic target, was recognized by 89.4% of the sera from primary syphilis patients (Table 2). Positivity to each of the 18 antigens was detected in 100% of the secondary syphilis sera, except for Tp0990, and in >90% of the early and late latent sera, with the exception again of Tp0990 (Table 2). When similar criteria for analysis (i.e., antigens that elicited a significant response in ≥85% of the sera, regardless of signal intensity) were applied to the control serum sample, IgG reactivity to the Tp0567-FlbB antigen remained elevated in the non-syphilis sera only, while no reactivity was found in any of the sera from periodontal disease patients. The 15 kDa lipoprotein (Tp0171-TpN15), also used in recombinant protein-based treponemal tests along with the TpN47 and TpN17 antigens, was not significantly recognized by any primary or secondary syphilis sera and only by 12.8% of the latent syphilis patients, with a mean SI only slightly above background (Table S1), suggesting that this antigen might be relatively less reactive and less useful compared to Tp0574-TpN47 or Tp0435-TpN17 to achieve early diagnosis, despite being a highly specific antigen for this pathogen. Additional studies will be needed to evaluate the validity of Tp0768-TmpA, Tp0567-FlbB, and Tp0319-TmpC as diagnostic antigens, as a subset of the non-syphilis sera also recognized these targets, particularly Tp0567-FlbB.

**TABLE 2:** Antigen eliciting an IgG-positive response in ≥85% of pre-treatment sera

Antigen	Annotated function^*a*^	% reactive/intensity^*c*^			
		All sera^*b*^	I syphilis^*d*^	II syphilis^*d*^	L syphilis^*d*^
Tp0768	TmpA lipoprotein	97.5/4.55	97.3/4.07	100/5.18	98.5/4.83
Tp0567	FlbB flagellar protein^*f*^	97.5/3.85	97.3/3.80	100/3.99	97.1/4.43
Tp0319	TmpC lipoprotein	97.5/4.16	97.3/3.58	100/4.87	97.1/3.94
Tp0574	TpN47/47 kDa lipoprotein	96.7/3.99	94.7/3.28	100/4.92	98.5/4.35
Tp0259	LysM/peptidoglycan-binding domain-containing protein	96.7/3.91	92.1/2.99	100/4.45	100/4.44
Tp0684	MglB/glucose-galactose binding lipoprotein	95.9/3.99	92.1/3.13	100/4.43	98.5/4.47
Tp0772	Hypothetical protein	95.9/4.45	92.1/3.76	100/5.18	98.5/4.87
Tp0435	TpN17/17 KDa lipoprotein	95.9/4.37	89.4/3.46	100/4.97	100/4.90
Tp0971	TpD/iron transporter lipoprotein	93.4/3.15	86.8/2.47	100/3.65	97.1/3.50
Tp0453	Concealed OMP	93.4/3.48	84.2/2.71	100/3.92	98.5/3.95
Tp0486	Hypothetical protein/Borrelia-like antigen p83/100	91.8/3.78	81.5/2.96	100/4.85	95.7/4.19
Tp0727	FlgE/flagellar hook protein	90.9/3.21	76.3/2.58	100/3.84	97.1/3.51
Tp0369	BamD/OMP assembly factor	89.3/2.75	81.5/2.36	100/3.42	91.4/2.92
Tp0433	Arp protein	88.5/2.99	73.6/2.36	100/3.44	95.73.35
Tp0625	Hypothetical protein	86.8/3.41	71.0/2.32	100/4.01	97.1/3.93
Tp0326	BamA/ OMP assembly factor	86.0/3.25	65.7/2.63	100/4.41	97.1/3.70
Tp0821	NlpA lipoprotein	85.2/2.60	71.0/2.40	100/3.60	92.8/2.89
Tp0990^*e*^	Putative membrane protein	85.2/2.04	81.5/1.99	87.2/1.93	88.5/2.11

^*a*^ Based on the annotation of the Nichols (NC_021490.2) and SS14 strain1.98 (NC_021508.1) genomes.

^*b*^ Positivity at enrollment (pre-treatment).

^*c*^ Mean intensity.

^*d*^ I: primary; II: secondary; L: latent (early, late, and latent of unknown duration).

^*e*^ NH_2_-terminal fragment (aa MPSA-HPND).

^*f*^ Based on the same criteria used to identify Table 2 IgG-reactive antigens, Tp0567 was recognized by ≥85% of the non-syphilis control sera.

When the IgA reactome was filtered for antigens that elicited a significantly positive signal in roughly half of the pre-treatment sera, seven antigens were identified. Only two, however, Tp0567-FlbB and Tp0768-TmpA, induced a relatively strong response in most of the sera at every stage analyzed (Table 3), although only Tp0567-FlbB was recognized by all secondary syphilis sera. No antigen was, however, recognized by the control sera using the same selection criteria.

**TABLE 3:** Antigen eliciting an IgA-positive response in roughly half of pre-treatment sera

Antigen	Annotated function^*a*^	% reactive/intensity^*c*^			
		All sera^*b*^	I syphilis^*d*^	II syphilis^*d*^	EL/LL/U syphilis^*d*^
Tp0567	FlbB flagellar protein	86.0/2.13	81.5/2.19	100/2.67	87.1/2.07
Tp0768	TmpA lipoprotein	70.4/1.68	60.5/1.41	87.5/2.03	74.2/1.81
Tp0772	Hypothetical protein	59.8/1.24	47.3/1.05	62.5/1.51	68.5/1.36
Tp0435	TpN17/17 kDa lipoprotein	58.1/1.25	42.1/0.97	75.0/1.30	68.5/1.43
Tp0727	FlgE/flagellar hook protein	53.2/1.34	42.1/1.31	75.0/1.68	57.1/1.34
Tp0445	PfpI family protein	51.6/0.99	47.3/0.98	37.5/0.90	57.1/1.00
Tp0486	Hypothetical protein/*Borrelia*-like antigen p83/100	47.5/1.25	34.2/0.99	44.4/1.11	55.7/1.44

^*a*^ Based on the annotation of the Nichols (NC_021490.2) and SS14 strain (NC_021508.1) genomes.

^*b*^ At enrollment (pre-treatment).

^*c*^ Mean intensity (in AU).

^*d*^ I: primary; II: secondary; EL/LL/U: early latent, late latent, and latent of unknown duration.

**Fig 3:**
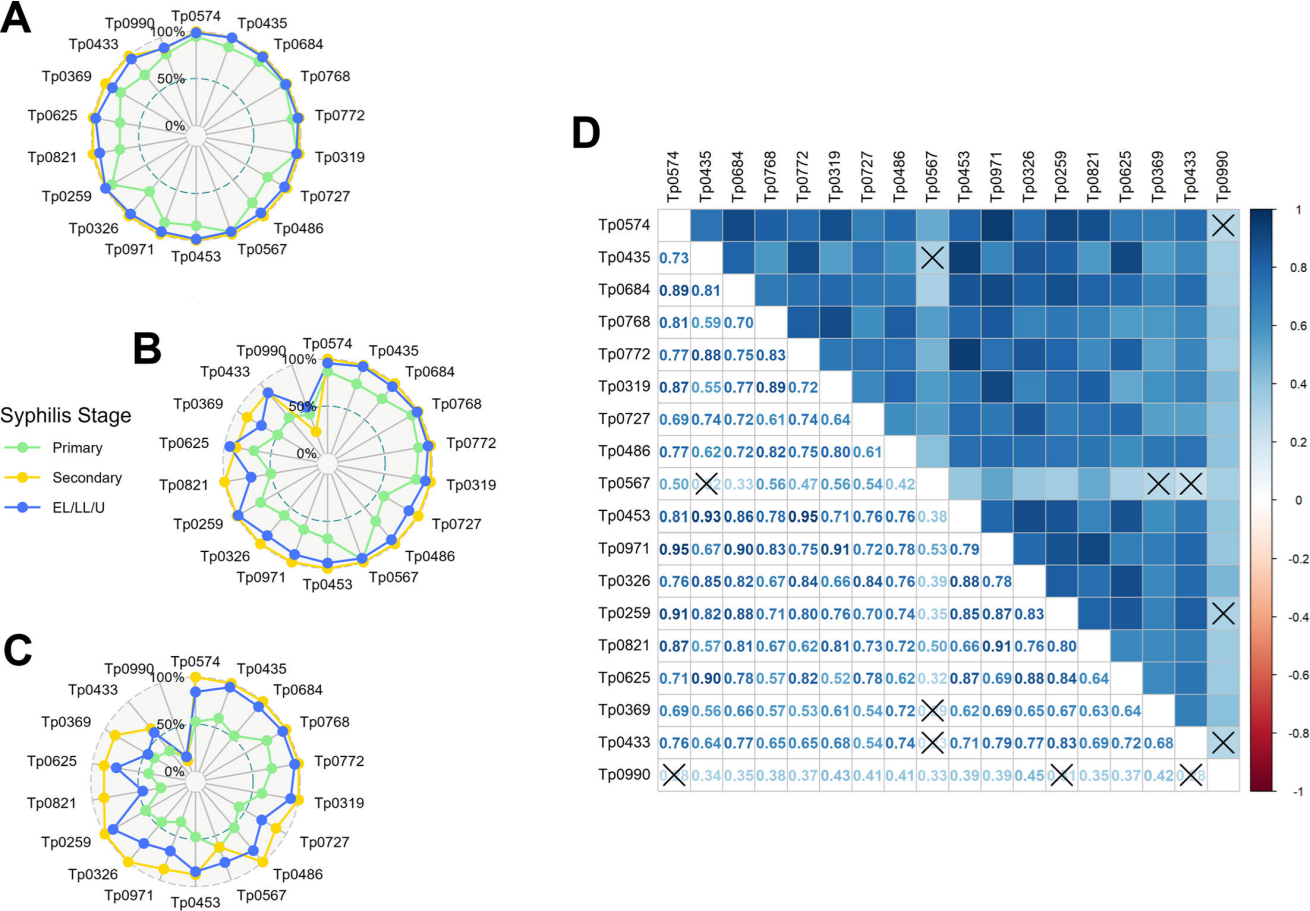
Serorecognition and correlation levels in pre-treatment primary sera between immunodominant antigens in the array. (**A–C**) The radar plots show the seroprevalence at enrollment (pre-treatment sera) of each antigen (% of positive responders) within patients grouped by syphilis stage at varying seropositivity cutoffs: (**A**) seropositive (≥1.0, or 2-fold over the background), (**B**) high reactivity (≥2.0, or 4-fold over the background), and (**C**) very high reactivity (≥3.0, or 8-fold over the background). Green: primary syphilis; gold: secondary syphilis; and blue: early latent and late/unknown latent (“EL/LL/U”). (**D**) Correlation analysis shows the linear correlation among the 18 most highly reactive antigens in pre-treatment primary sera. Pearson correlation coefficients are shown in the bottom diagonal and expressed in the gradient next to the graph and reported for each comparison in Table S9. Squares marked with an “X” identify correlations lacking significance.

In addition, we also analyzed which antigens were associated with the highest signal in pre-treatment primary syphilis sera, with the rationale that such antigens could facilitate early detection of a new infection. The mean reactivity to Tp0768-TmpA (4.07), Tp0567-FlbB (3.80), Tp0772 (3.76), and Tp0319-TmpC (3.58) in these sera was generally higher at collection (pre-treatment) compared to all other antigens (Table 2), albeit the quantification of differences in antibody binding between proteins on microarray has limitations due to difficulties in assessing how much target antigen is produced during the *in vitro* transcription and translation (IVTT) process. Serorecognition between subject groups, in contrast, is valid in all comparisons, and in pre-treatment sera, recognition of antigens at the seropositivity threshold was lower in primary cases than secondary and early and late latent cases as well as latent cases of unknown duration by more than 20% for Tp0326 (66%, 100%, and 97%, respectively), Tp0821 (71%, 100%, and 93%), Tp0625 (71%, 100%, and 97%), Tp0433 (74%, 100%, and 96%), and Tp0727 (76%, 100%, and 97%) (Fig. 3A and Table S6). Notably, Tp0574, Tp0768, Tp0319, and Tp0567 differed by only 5% or less between syphilis stages. Greater variation between stages was observed in levels of serorecognition at high signal intensities (SI) of 2.0, or 4 times the background (Fig. 3B and Table S7), and very high SI of 3.0, or 8 times the background (Fig. 3C and Table S8). These illustrate antigens that may be useful for differential staging diagnosis, such as Tp0821 at SI ≥ 2.0 (50%, 100%, and 71%) or Tp0326 at SI ≥ 3.0 (45%, 100%, and 74%).

In the pre-treatment sera, we analyzed how well the reactivities to each of the 18 highly recognized targets (Table 2) correlated with each other by calculating Pearson correlation coefficients, with the rationale that antigens whose reactivities correlated during infection could be paired in diagnostic tests. The results showed that in general, a significant positive correlation was seen for most antigens (Fig. 3D). In more detail, reactivity to the most highly recognized antigen, Tp0768-TmpA, strongly correlated with that of Tp0319-TmpC (ρ = 0.89) and Tp0486 (ρ = 0.82). Tp0319-TmpC signal also strongly correlated with Tp0971-TpD (ρ = 0.91). Reactivity to the immunodominant lipoprotein Tp0574-TpN47 correlated with that of Tp0971-TpD (ρ = 0.95) and Tp0259-LysM (ρ = 0.91), while Tp0435-TpN17 most strongly correlated with that of the hypothetical proteins Tp0453 (ρ = 0.93), Tp0625 (*P* = 0.90), and Tp0772 (ρ = 0.88) (Fig. 3D and Table S9).

**Fig 4:**
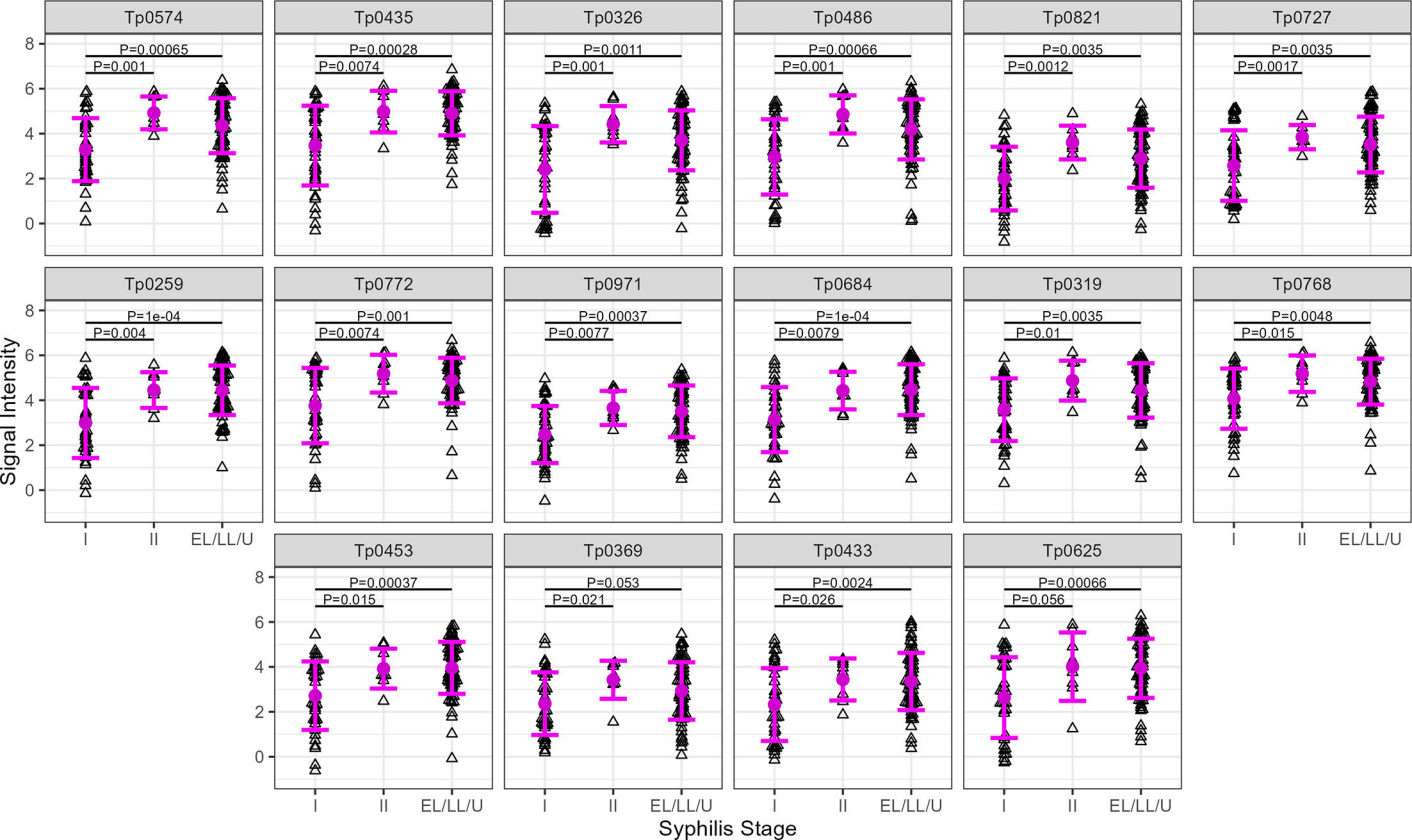
IgG reactivity to highly reactive *T. pallidum* antigens in pre-treatment sera by syphilis stage. The bee-swarm plots show IgG binding levels in primary (*N* = 38), secondary (*N* = 8), and early latent, late latent, or latent of unknown duration (*N* = 70) patients at pre-treatment. Group means and standard error of mean (SEM) bars are shown in magenta. False discovery rate-adjusted *P*-values from univariate Student’s *t*-tests between syphilis stage groups are shown above each comparison with the primary syphilis group. Abbreviations: I, primary syphilis; II, secondary syphilis; EL/LL/U, early latent, late latent, or latent syphilis of unknown duration.

In the pre-treatment sera, there were significantly lower IgG levels in primary syphilis patients than in secondary or early latent/late latent/unknown duration (EL/LL/U) patients against 15 of the 18 antigens in Table 2 (Fig. 4 and Table S9). No differences were found between secondary and early latent/late latent/unknown duration syphilis IgG levels. HIV status and history of syphilis also had no significant differential reactivity in pre-treatment sera (Fig. S3).

The same analysis performed for IgA showed that only Tp0326-BamA had lower primary syphilis IgA levels in comparison with early latent/late latent/unknown duration syphilis sera, but not in comparison to secondary syphilis sera (Fig. S4). No significant differences in IgA levels between syphilis stages were seen for the two most recognized antigens by IgA (Tp0768-TmpA and Tp0567-FlbB) or the immunodominant lipoproteins Tp0435-TpN17 and Tp0574-TpN47 (Fig. S4).

### Longitudinal analysis of reactivity

**Fig 5:**
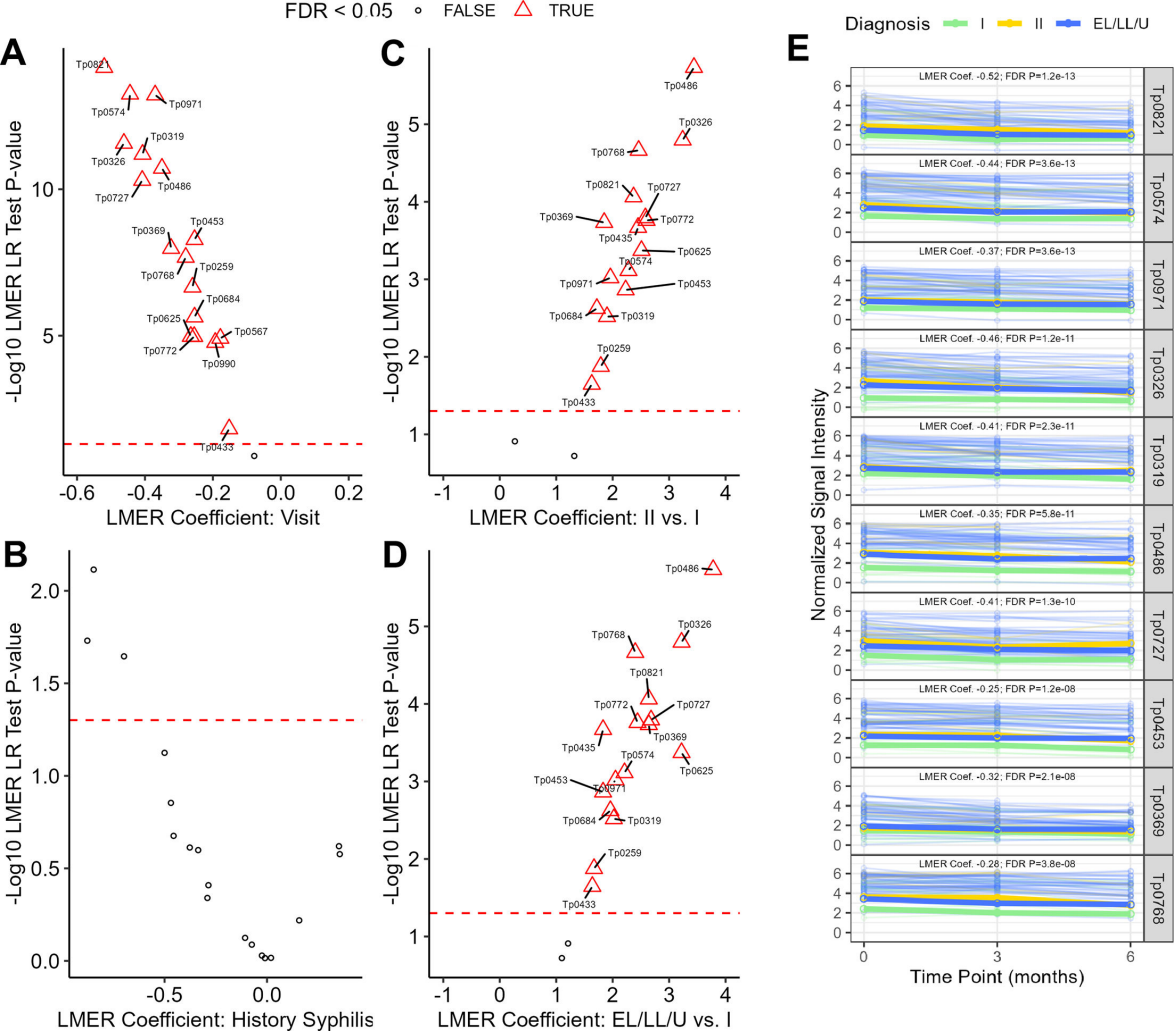
Longitudinal reactivity to selected *T. pallidum* antigens using LMER. LMER models estimating the longitudinal trajectory of antibodies adjusted for age, gender, MSM, sex worker status, HIV, presence of lesions, history of syphilis, and diagnosis of syphilis stage. (**A-D**) The volcano plots show the LMER coefficient of the effect of covariates on antibody levels on the *x*-axis and inverse log_10_ *P*-value from likelihood ratio (LR) tests of LMER null vs. full models on the *y*-axis. Responses associated with antibody levels after correction for the false discovery rate (FDR) are shown in red labeled triangles. The horizontal dashed line represents an unadjusted *P*-value of 0.05. (**E**) The spaghetti plots show individual subject antibody trajectories over follow-up from admission (month 0) to 3 and 6 months post-admission and treatment. The top 10 antigens by LMER *P*-value for the visit are shown. Lines are colored by diagnosis stage, and bold lines represent the stage means at each time point. A total of 64 samples were analyzed pre-treatment (month 0), 50 samples for the 3-month visit, and 43 samples at the 6-month visit, as not all patients provided a sample at follow-up time points.

The longitudinal analysis of samples showed a significant decrease in IgG antibody reactivity for 17 antigens from pre-treatment to 6 months post-treatment (Fig. 5A). The most significant declines were seen for Tp0821 (β = −0.52, *P* = 1.02E−14), Tp0574 (β = −0.44, *P* = 6.01E−14), and Tp0326 (β = −0.46, *P* = 2.84E−12). The estimated overall reduction of these three antibody levels is approximately 10% per month after treatment administration. There were no significant effects of the history of syphilis on longitudinal antibody levels (Fig. 5B and Table S11). The effect of staging on antibody levels was similar to the profile of antigen serorecognition seen in Fig. 3A through C, with secondary syphilis and latent syphilis stages having higher antibodies than primary syphilis (Fig. 5C and D, respectively, and Table S11). The trajectories (on a log_2_ scale) of antibody levels to the 10 most significant antigens by linear mixed effects regression (LMER) *P*-value for visit are shown in Fig. 5E. There were no significant effects also when covariates such as gender, sexual orientation, sex worker status, HIV infection, or presence of lesions were considered, but there was a small effect of increasing antibodies with age (β ≈ 0.05, or approximately 1% per year of age) (Fig. S5 and Table S12).

The same analysis performed for IgA antibodies identified 13 antigens with a significant decrease in reactivity from pre-treatment to 6 months post-treatment (Fig. S6 and Table S13). The most significant declines were seen for Tp0326 (β = −0.19, *P* = 3.3E−9), Tp0435 (β = −0.21, *P* = 4.3E−9), and Tp0768 (β = −0.4, *P* = 4.8E−9). The estimated overall reduction of these three antibody levels is approximately 14%, 16%, and 32% per month, respectively. Like for IgG antibodies, there were no significant effects of history of syphilis on longitudinal antibody levels or covariates such as gender, sexual orientation, or sex worker status, HIV infection, or the presence of lesions (Fig. S6), but there was a small effect of increasing antibodies with age for nine targets (β ≈ 0.02–0.04, or approximately 1.4%–2.8% per year of age) (Fig. S6 and Table S13). The effect of staging showed that Tp0433 and Tp0486 antibody levels were higher in secondary and early latent/late latent/unknown duration sera compared to primary sera (Fig. S6).

**Fig 6:**
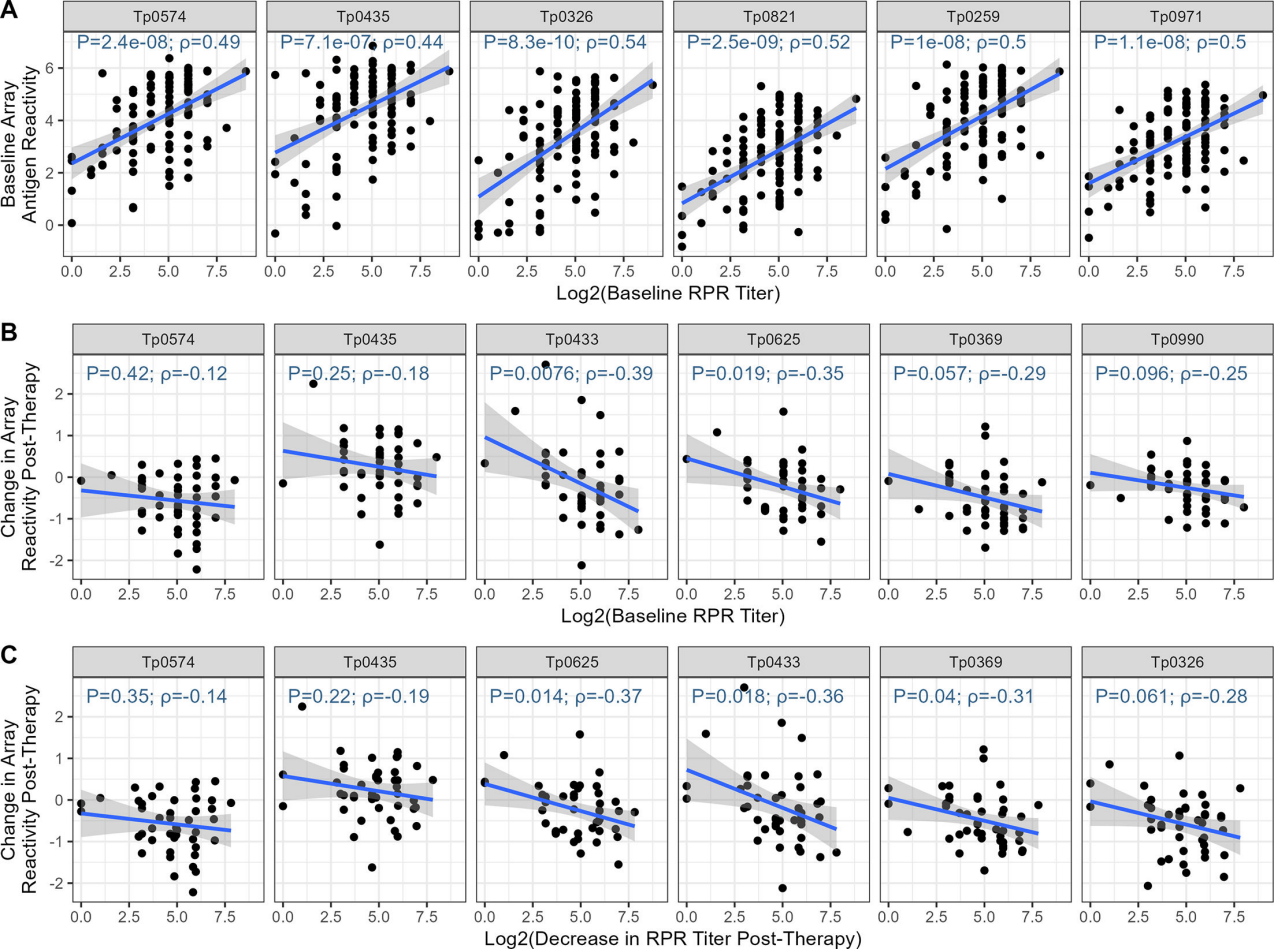
Correlation between reactivity of selected antigens in pre-treatment sera and RPR titer, and between changes in reactivity at 3 months post-treatment and decrease in RPR titer. (**A**) The scatter plot shows pre-treatment IgG levels of Tp0574-TpN47, Tp0435-TpN17, and the four most significant antigens by linear regression (*y-*axis) in association with pre-treatment RPR titers (*x*-axis). (**B**) Change in IgG SI post-treatment from pre-treatment to 3 months, or “Deltas” (*y*-axis), is shown with pre-treatment RPR titer (*x*-axis). (**C**) IgG Deltas are shown with the decrease in RPR titer from pre-treatment to 3 months. Not enough RPR data points were available at visit 2 (6 months post-treatment) to conduct this analysis; blue lines represent a linear regression fit with 95% confidence bands. *N* = 114 for panel **A**; *N* = 45 for panel **B**; and *N* = 44 for panel **C**. For the RPR titers, a constant of 1 was added to facilitate conversion, and all RPR values are shown on the log2 scale. Log2RPR values of 1–8 represent 1:1–1:128. Linear regression *P*-values and Pearson’s correlation coefficients (ρ) are reported for each antigen in Table S14.

Finally, to further explore the decrease in reactivity to selected antigens post-treatment, pre-treatment IgG-binding levels to the highly reactive antigens (Table 2) were analyzed for correlation with RPR titers. All antigens except for Tp0567 and Tp0990 had a significant positive association with pre-treatment RPR titers by linear regression; however, only a moderate correlation was observed (Pearson’s correlation coefficient range: ρ = 0.34–0.54) (Table S14). Tp0574-TpN47, Tp0435-TpN17, and the four most significant antibody responses associated with pre-treatment RPR titer are shown in Fig. 6A. When comparing the change in antibody levels in pre-treatment sera and at 3 months post-treatment, there was a negative association with pre-treatment RPR titers for Tp0433 and Tp0625, showing that patients with higher pre-treatment RPR titers had greater decreases in antibody levels to these two antigens, while other antigens were nonsignificant after FDR correction, and correlations were low to moderate in all cases (Fig. 6B). When we investigated whether the change in antibody levels mirrored the change in RPR titers post-therapy, Tp0625 and Tp0433, with the addition of Tp0369, were significantly associated with RPR titers (Fig. 6C). The heterogeneity in individual patient responses post-therapy suggests that some individuals have more persistent antibody responses to some antigens than others.

## DISCUSSION

The goal of this study was to analyze antibody reactivity to virtually all *T. pallidum* antigens in a relatively large group of sera collected pre- and post-treatment from syphilis patients to identify highly reactive targets and antibody dynamics over time in response to treatment. These data could improve the ability of treponemal serological tests to perform early diagnosis or to facilitate disease management post-treatment. In part, this goal was also pursued in 2006 by Brinkman and colleagues (31), who generated a 908-protein array based on the first sequenced Nichols strain genome (35) by expressing and purifying each *T. pallidum* antigen in *Escherichia coli*, and then testing this earlier array in an ELISA format with sera from patients with primary (*n* = 2), secondary (*n* = 9), and early latent (*n* = 5) infection collected at diagnosis, but without post-treatment follow-up sera. Compared to this early study (31), where the authors identified 38 reactive antigens (4.1% of the total number analyzed and 3.6% of the pathogen’s proteome), we can more confidently conclude that during infection, the fraction of the *T. pallidum* proteome that elicits a significant IgG response is closer to ~10%, highlighting the importance of analyzing a larger cohort of patients for immunological studies. Of the 38 antigens discovered by Brinkman et al. (31), 85% were found to be reactive also in our study. Exceptions included antigens such as Tp0292 (a *T. pallidum* OmpA homolog), Tp0767-FusA2, Tp1015-NusB, and the hypothetical proteins Tp0750 and Tp0956, which were recognized by a subset of the sera used in a study by Brinkman et al. (31) but did not meet the positivity criteria of our statistical analysis. Despite such differences, both Brinkman’s study and ours highlighted the immunodominance of lipoproteins such as Tp0768-TmpA, Tp0684-MglB, and Tp0319-TmpC during infection, which represent possible diagnostic candidates in addition to the widely used Tp0435-TpN17 and Tp0574-TpN47. Detection of reactivity to a subset of control sera to Tp0768-TmpA and Tp0319-TmpC, however, suggests that their specificity as diagnostic targets might not be as elevated as it is for other antigens, although the number of control sera tested was small. In a previous study with the same array used here, we evaluated the development of the IgG and IgM response to the *T. pallidum* proteome in long-term (90 days) infected New Zealand White rabbits that were inoculated intradermally (ID) either with the *T. pallidum* Nichols or SS14 strain (36) and from which the serum was collected longitudinally every 10 days post-infection. In this earlier study, rabbits were found to develop a significant IgG reactivity to a total of 351 proteins, corresponding to roughly one-third of the pathogen’s proteome. Also, in the rabbit model of experimental syphilis, the most highly reactive antigens were lipoproteins, and all the targets recognized by patient sera (this study) were reactive in sera from infected animals (36). In spite of these similarities, however, there is indeed a remarkable difference in the number of positive targets seen in infected patients vs. experimentally infected rabbits. Such a discrepancy could be attributed to the relatively high *T. pallidum* inoculum used in rabbits (six million total treponemal cells, subdivided into six injection sites) compared to the smaller pathogen burden patients are likely exposed to in a natural infection. Given that the mouse model of infection is increasingly being adopted in the field, current analyses are ongoing to understand the immunity that develops in several laboratory strains of these animals after long-term infection. Although one could hypothesize that the extent of the humoral response in mice resembled that in rabbits, the fact that, unlike rabbits, mice do not develop symptoms post-inoculation might lead to important differences in how mice respond to infection and help elucidate further in which areas of syphilis research these animals are to be increasingly adopted to elucidate disease pathogenesis in a way that relates to human disease.

One of the most evident differences between the humoral immunity resulting from natural infection compared to the one in infected animals is that almost the entirety of the *T. pallidum* repertoire of putative OMPs was recognized by rabbit sera collected at the end of the experiment, although at low intensity. In contrast, patient sera only significantly recognized four OMPs (Tp0326-BamA, Tp0621-TprJ, Tp0117-TprC, and Tp0131-TprD). It is established that long-term infected rabbits develop partially protective immunity, which leads to attenuation of disease manifestations upon reinfection (37, 38), and clinical studies have shown that patients who experience a syphilis episode are more likely to be asymptomatic if another episode occurs (39–42). Altogether, these findings suggest that an effective vaccine for syphilis may be attainable if the right *T. pallidum* surface antigens are targeted. Because evidence exists that antibodies to *T. pallidum* surface-exposed OMPs are mediators of protective immunity (43), the identification of the OMP that preferentially elicits a host response during infection, such as the ones recognized by patient sera in this study, could provide important clues as to which antigens to include in an experimental vaccine design. The sera used in this study are currently being investigated via PhIP-Seq using a phage oligonucleotide library representing 92,743 unique *T. pallidum*-translated peptides from a total of 608 unique *T. pallidum* strains comprising all three *T. pallidum* subspecies, collected from 38 countries and described in more detail elsewhere (44). These experiments will allow us to identify which epitopes, particularly in reactive OMPs, are being recognized in these individuals. In turn, these sequences will be considered as components of the chimeric antigens currently being developed as an experimental syphilis vaccine.

Although many clinical studies performed in the past recognized Tp0171/TpN15 as a useful serodiagnostic antigen (45–49), it is intriguing that this antigen was overall poorly recognized by patient sera in our study, despite being remarkably conserved across *T. pallidum* strains and subspecies. This result, however, is consistent with the study by Brinkman et al., where Tp0171 was not among the 38 antigens that were significantly recognized by their patient sera. Furthermore, seropositivity to TpN15 developed only after day 40 post-inoculation in infected animals (36). Furthermore, our internal controls supported the expression and proper deposition of TpN15 on the proteomic array. Although we do not have a plausible explanation for this discrepancy between our study and others, we concluded that the use of TpN15 for early diagnosis could be revisited, given that we identified several additional antigens worth investigating to increase the sensitivity of treponemal tests (Table 2).

In our previous pre-clinical study, we also explored the IgM response to *T. pallidum* antigens in infected animals (36) in addition to the IgG response. Our results showed that only 13 antigens were recognized by IgM in long-term infected rabbit sera, which prompted us to evaluate the IgA response in patient sera in this study instead. IgA monomers are the second most prevalent antibody class in serum and the predominant one in mucosal exudates, where they are present as dimers (50). Detection of serum IgA is considered a possible serological marker for a variety of infections, given that they only persist as long as antigenic stimulation persists and serum IgA is metabolized approximately five times faster than IgG (51, 52). Currently, there are three reports on syphilis and IgA detection to perform diagnosis (53–55). The study by Schmitz et al. (55) used western blot technology with whole *T. pallidum* lysates to assess the presence of IgA antibody in either cord blood or serum from infants with (or at risk of) congenital syphilis. Rodriguez et al. (53) used recombinant Tp0435-TpN17, Tp0574-TpN47, and Tp0768-TmpA as test antigens, while Pham et al. (54) used Tp0171-TpN15, Tp0435-TpN17, and Tp0574-TpN47. All studies concluded that IgA detection had potential utility in a clinical setting to diagnose syphilis or congenital syphilis, although further investigation was needed. While Rodriguez et al. (53) only focused on pre-treatment sera, Pham et al. (54) analyzed 154 sera from active syphilis patients (TPHA/RPR double positive) and 153 sera from treated individuals (still TPHA positive, but RPR negative) and reported that 96% of the active syphilis sera were IgA-positive, while a significant proportion of post-treatment sera (40%) were IgA-negative, albeit still IgG positive. Although the pre-treatment and post-treatment sera were not longitudinally collected from the same patients, these data suggested seroreversion upon treatment. Of the antigens explored in these two previous studies, only Tp0435-TpN17 and Tp0768-TmpA appeared to be suitable targets here, as they were recognized by 58.4% and 70.1% of pre-treatment sera. In our study, in addition to Tp0768-TmpC, Tp0567-FlbB followed by Tp0727-FlgE, Tp0435-TpN17, or Tp0772 would seem to be plausible choices to try and develop an IgA-based assay. Furthermore, following treatment, our data show that the IgA SI also declined for most of the above antigens (Fig. S6). Because, like IgM, IgA does not cross the placenta, the role of IgA antibodies could also be explored in the diagnosis of congenital syphilis as done in the past to attempt to use IgA antibodies for the diagnosis of congenital toxoplasmosis (56). To this end, in newborns for whom there is reason to suspect transplacental transmission, the serum collected via heel stick before breastfeeding starts might be the specimen type worth pursuing, to expand on the work initiated by Schmitz et al. back in the early 90s (55). This study, in fact, concluded that about 66.6% of the sera from infants with (or most at risk for) congenital syphilis were IgA-positive. A limitation of this study, like some of the others conducted so far to detect IgA antibodies in syphilis patients, is that samples did not undergo depletion of IgG antibodies, which could compete for antigen binding, thus potentially decreasing the signal from IgA. IgG interference, in our case, would likely completely ablate the IgA microarray signal at near-saturation levels of specific IgG. This instance, however, was not observed in the study or during microarray QC, although it remains unknown if IgA levels would have been higher if IgG were depleted. Future studies will address this potential pitfall.

In conclusion, the proteomic array that we developed in combination with a substantial number of well-characterized sera collected pre- and post-therapy from syphilis patients allowed us to identify a few highly reactive antigens that could be used to increase the sensitivity of current serological assays, although none of the antigens analyzed here could effectively achieve disease staging and monitor response to treatment via either IgG or IgA detection.

## MATERIALS AND METHODS

### Study sites and population

Study participants were recruited between 2019 and 2021 from five sexual health clinics. Three of these were in the Lima metropolitan area (Independencia, San Juan de Lurigancho, and Barranco districts, respectively), one was in the Callao region, and one was in the city of Pucallpa (in eastern Peru). All centers provide testing and care for STIs and are attended by MSM, sex workers, and transgender women. To be enrolled, participants had to be 18 or older and newly diagnosed with active syphilis, with documentation of prior syphilis testing and/or treatment at the recruitment site, availability to return to the site for follow-up visits at 3 and 6 months post-treatment, and openness to provide the required biological specimens. Active syphilis was determined by clinical (having primary or secondary syphilitic lesions) and/or serological criteria (new 2-fold RPR titer rise since the last lipoidal test or new seroconversion based on the rapid treponemal test performed at enrollment; see below). Diagnoses of clinical stage of syphilis among those with active syphilis were determined by trained physicians based on serological and clinical criteria based on current international and local guidelines (57, 58): primary syphilis was diagnosed if the patient had primary lesions (chancre), secondary syphilis was diagnosed if patients had secondary lesions, early latent syphilis was diagnosed if patients had no symptoms and previous RPR or treponemal tests within 12 months as proof of recent exposure to *T. pallidum*, while latent syphilis of unknown duration was called in the absence of lesions and no RPR or treponemal testing within 12 months on clinical records. Two groups of patients were recruited in this study: individuals without a history of syphilis and individuals with repeat infection. Participants were categorized as having no history of syphilis if they had a newly reactive RPR at enrollment, a positive TPPA test, and a documented negative treponemal antibody test taken within the prior 12 months. Repeat syphilis infection was defined by a new 4-fold titer increase or newly reactive RPR with a previously positive RPR test titer (≥ 1:8) or a documented positive treponemal antibody test. A minority group of individuals with unknown syphilis history at the recruitment site but presenting syphilitic-like genital lesions upon examination were also enrolled. Subjects meeting eligibility criteria and willing to participate signed an informed consent form and received appropriate reimbursement for providing samples and transportation costs. Cohort data are reported in Table 1. Disease staging was performed according to the CDC guidelines (https://ndc.services.cdc.gov/case-definitions/syphilis-2018/). Of all enrolled patients, only those who achieved serological cure (4-fold decrease in lipoidal antibody titers) were used in this study. Serum specimens from syphilis-negative patients (n = 11), and from patients with rapidly progressing periodontitis (RPP; n = 5) and localized juvenile periodontitis (LJP, n = 5) were also used as negative controls for the determination of specificity and/or cross-reactivity of anti-syphilis antibody responses.

### Specimen collection and testing

At the clinics, the serum extracted from all patients with or without syphilis by centrifugation after venipuncture was tested on-site with the Abbott Determine Syphilis TP test (Abbott, Chicago, IL; cat. #7D2453), a WHO-prequalified 10-min point-of-care test that detects IgM and IgG treponemal antibodies to recombinant treponemal antigens Tp0171, Tp0435, and Tp0574, and shown in previous studies to have high sensitivity (88%; 95% confidence interval [CI], 81%–96%), the lowest rate of indeterminate tests (0.8%), and 100% specificity (59). The RPR test (Wiener Laboratorios; Santa Fe, Argentina; cat. #1120007) was used for lipoidal antibody screening. Samples were also tested for HIV either with the Determine HIV-1/2 Ag/Ab Combo (Abbott; cat. #7D2648) or the HIV 1/2 Ab Plus Combo Rapid Test (CTK Biotech, Poway, CA; cat. #R0011C). Serum aliquots were then reserved for analysis using the antigen array described here. All samples were properly stored and shipped frozen to the Laboratory of Sexual Health at Universidad Peruana Cayetano Heredia for additional testing and long-term storage at −80°C until use. Treponemal testing for syphilis was done using the Fujirebio (Tokyo, Japan; cat. #234952) TPPA assay, irrespective of the RPR result attained at the clinics. The results of HIV and syphilis tests were communicated to participants along with appropriate counseling. Appropriate further clinical evaluation for enrollment in the HIV treatment program at each site was provided, as necessary. All syphilis cases were treated on-site using antibiotics recommended by the CDC STI Treatment Guidelines (60). Sera from de-identified periodontal disease patients were originally provided by Dr. Richard Riviere (Oregon Health Sciences University, Portland, OR).

### Array construction and probing

A core proteome was identified using the genome sequences of the Nichols (NC_021490.2) and SS14 (NC_021508.1) strains as annotated by the NCBI Prokaryotic Genome Annotation Pipeline (PAGAP) (https://www.ncbi.nlm.nih.gov/refseq/annotation_prok/) and as described in previous studies (36). Pan-proteome arrays were fabricated containing 1,040 full-length or fragmented recombinant proteins representing a total of 1,009 genes (Table S15). Of these, 986 were genes encompassing the core proteome shared by the two strains, 10 were genes encoding for Nichols-specific proteins, and 13 were specific for the SS14 strain. Each open reading frame (ORF) sequence was PCR-amplified, inserted into the vector pXT7 by recombination in *E. coli* to establish a library of partial or complete coding sequences. Proteins were expressed using a coupled *E. coli* cell-free IVTT system (Rapid Translation System, Biotechrabbit, Berlin, Germany, cat. #BR1400201) and spotted onto nitrocellulose-coated glass AVID slides (Grace Bio-Labs Inc., Bend, OR, cat. #GBL305278-20EA) using an Omni Grid Accent robotic microarray printer (Digilabs Inc., Hopkinton, MA). A small subset of targets not represented in the array due to failed cloning is reported in Table S16. The Tp0897-TprK putative OMP was the only antigen purposely omitted from the list of targets to clone due to its intra-strain antigenic hypervariability (61). Each expressed protein included a 5′ poly-histidine (His) epitope and a 3′ hemagglutinin (HA) epitope. Pan-proteome array chip printing and protein expression were quality-checked by probing random slides with anti-His and anti-HA monoclonal antibodies, fluorescently labeled and quantifying spot signals using a microarray scanner. As positive control antigens, six recombinant *T. pallidum* proteins previously used to construct a minimal proteomic array (62) were spotted onto the array at two different concentrations. Those controls included the known immunodominant lipoproteins Tp0435-TpN17, Tp0574-TpN47, and Tp0768-TmpA, known to be conserved in both strains, the putative OMPs Tp0117-TprC and Tp0131-TprD, and the amino-terminal fragment of the Tp0897-TprK antigen (aa 29–274/AQV–ALA) carrying two predicted conserved surface-exposed loops of this antigen already known to be targeted by the host response during infection (63).

Serum samples were diluted (1:100) and incubated on the array chips overnight at 4°C on a rocker. Bound IgG was detected with DyLight650-conjugated goat anti-human IgG (Bethyl, Fortis Life Sciences, Cat# A80-104D5), and IgA was detected with Cy3-conjugated goat anti-human serum IgA α chain (Jackson ImmunoResearch, Cat# 109-166-011). Washed and dried arrays were scanned, and the spot and background signal intensities (SI) were exported into the R package for statistical analysis.

### Statistical analysis

Spot SIs were adjusted for local background by subtraction, and values were floored to 1. Next, the data were normalized by dividing the protein spot values by the median of IVTT control spots (IVTT expression reactions with no *T. pallidum* ORFs), and values were log-transformed using the base-2 logarithm. Thus, normalized data represented the log2 signal-to-noise ratio, where a value of 0 represents a specific antibody SI equal to the background, 1.0 represents twice the background, 2.0 represents 4-fold over the background, and so forth. *T. pallidum* protein responses were classified as seropositive for SI of at least 1.0, or twice the background. Maximal SIs across all timepoints for each subject were calculated for each protein. A protein was classified as “reactive” if over 25% of the study population responded to the protein, i.e., 32 or more individuals with a seropositive response. A “high reactivity” threshold of 85% seroprevalence was used to examine a subset of reactive antigens. Individual antibody responses at pre- and post-treatment time points were visualized using the ComplexHeatmap package (64). The distributions of reactive antigen responses were visualized using error bar plots of all proteins. Overlap in reactivity to individual antigens between groups was visualized using the VennDiagram package. The association of protein physiochemical and functional features with antibody reactivity was analyzed using random forest (RF) for feature selection. RF models used protein features and classification of proteins IgG- and/or IgA-reactive, and were repeated 100 times, randomly resampling 90% of protein features. The percentage increase in mean square error (%incMSE) was used to determine variable importance. A significance cutoff of 5% was used to classify important variables, and the percentage of times each antigen was identified as an important variable was used to select features that were significantly associated with reactivity with a cutoff of at least 80% of RF models. Only protein features present in at least five antigens were included in the models. The association of pre-treatment antibody levels with age, sex, previous history of syphilis, presence of lesions, diagnosis of syphilis stage pre-treatment, HIV status, categorization as a sex worker, and categorization as men who have sex with men was estimated using ordinary least-squares (OLS) linear regression. The trajectory of antibodies following treatment was estimated using linear mixed effects regression, adjusting for multiple measures per individual and for fixed effects specified in the OLS linear regression models.

Differences between time points were visualized using volcano plots, and longitudinal trajectories of specific antibody responses were visualized using line plots with the ggplot2 package (65). Only reactive antigens were included in differential analysis, and *P*-values were adjusted for the FDR using the method described by Benjamini and Hochberg (66).

## SUPPLEMENTAL MATERIAL

Figure S1 mbio.00820-26-s0001.tif Overall IgG and IgA reactivity.

Figure S2 mbio.00820-26-s0002.tif Random forest analysis of protein features associated with antigen reactivity.

Figure S3 mbio.00820-26-s0003.tif IgG reactivity to highly immunogenic *T. pallidum* proteins.

Figure S4 mbio.00820-26-s0004.tif IgA reactivity to highly immunogenic *T. pallidum* antigens in pre-treatment sera.

Figure S5 mbio.00820-26-s0005.tif Effect of covariates on IgG antibody levels to selected *T. pallidum* antigens.

Figure S6 mbio.00820-26-s0006.tif Effect of covariates on IgA antibody levels to selected *T. pallidum* antigens.

Legends mbio.00820-26-s0007.docx Legends for the supplemental figures.

Supplemental Tables mbio.00820-26-s0008.xlsb Tables S1-S16.

ASM does not own the copyrights to Supplemental Material that may be linked to, or accessed through, an article. The authors have granted ASM a non-exclusive, world-wide license to publish the Supplemental Material files. Please contact the corresponding author directly for reuse.
