# Supplementary figures and images for: High-throughput profiling of the IgG and IgA response to the Treponema pallidum subsp. pallidum proteome in syphilis patients
Source: mBio. 2026 Jun 9;17(7):e00820-26. doi: 10.1128/mbio.00820-26 (PMC13343974; doi:10.1128/mbio.00820-26)

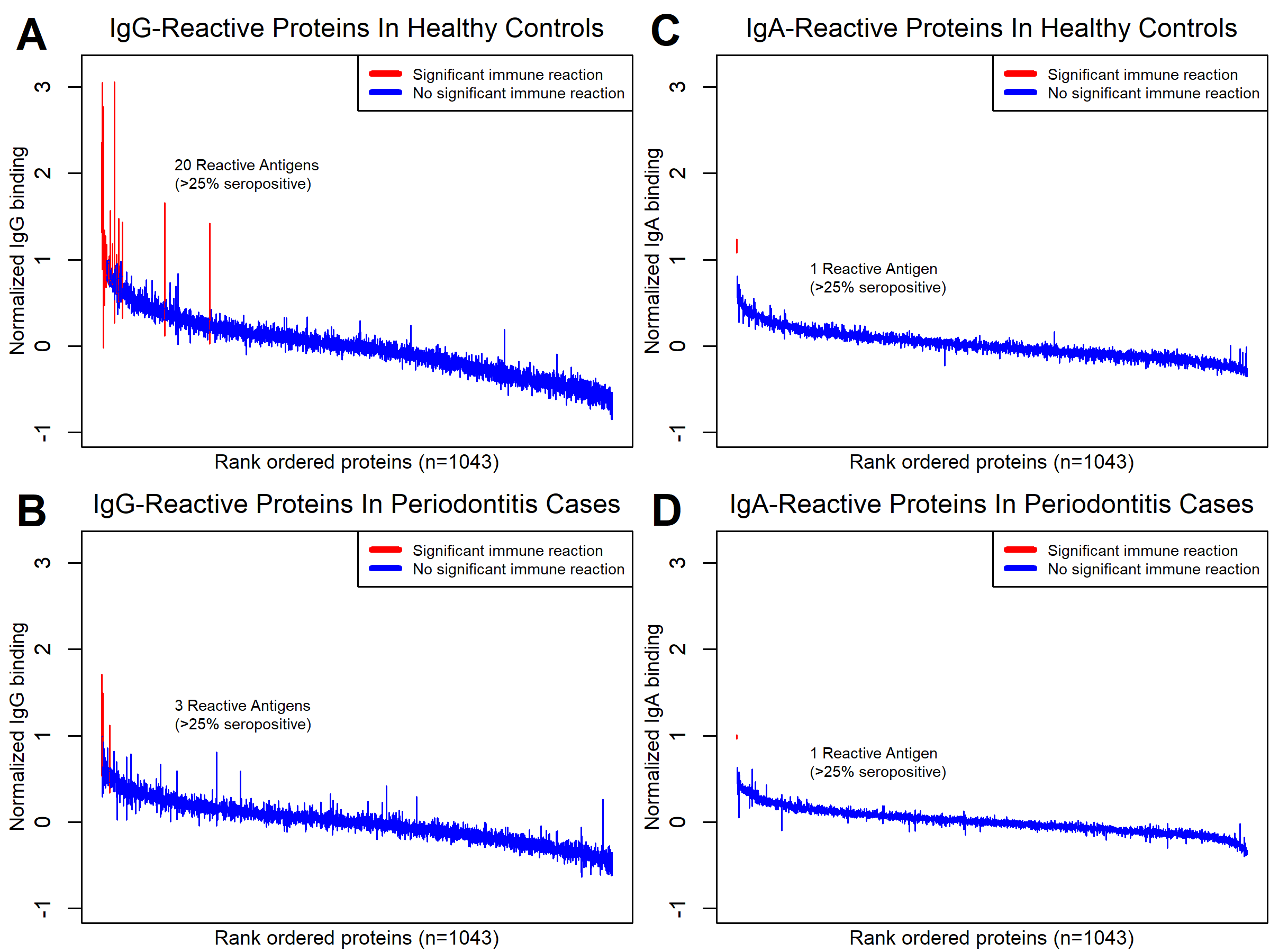

Supplement: Figure S1 — Overall IgG and IgA reactivity. [file mbio.00820-26-s0001.tif]

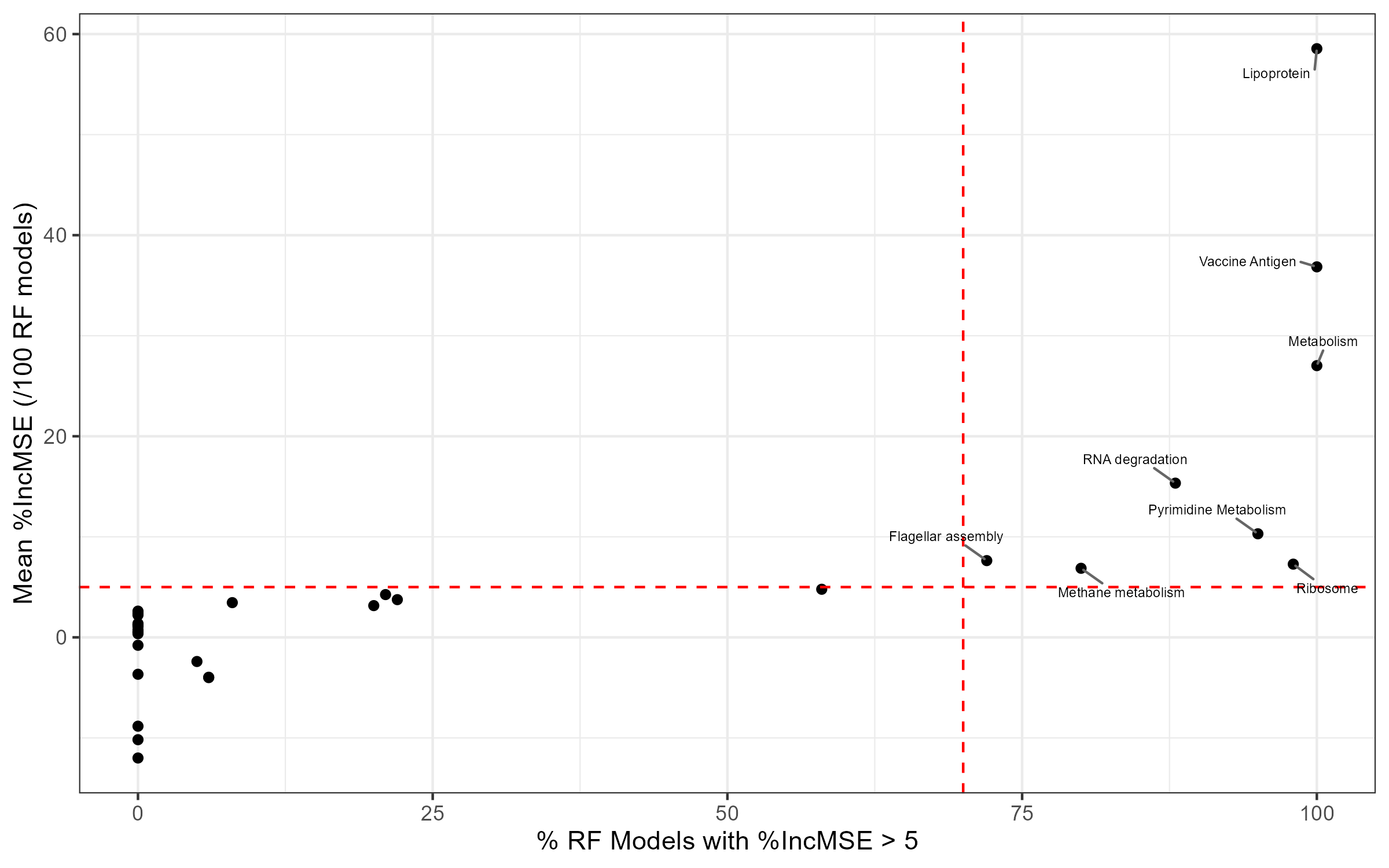

Supplement: Figure S2 — Random forest analysis of protein features associated with antigen reactivity. [file mbio.00820-26-s0002.tif]

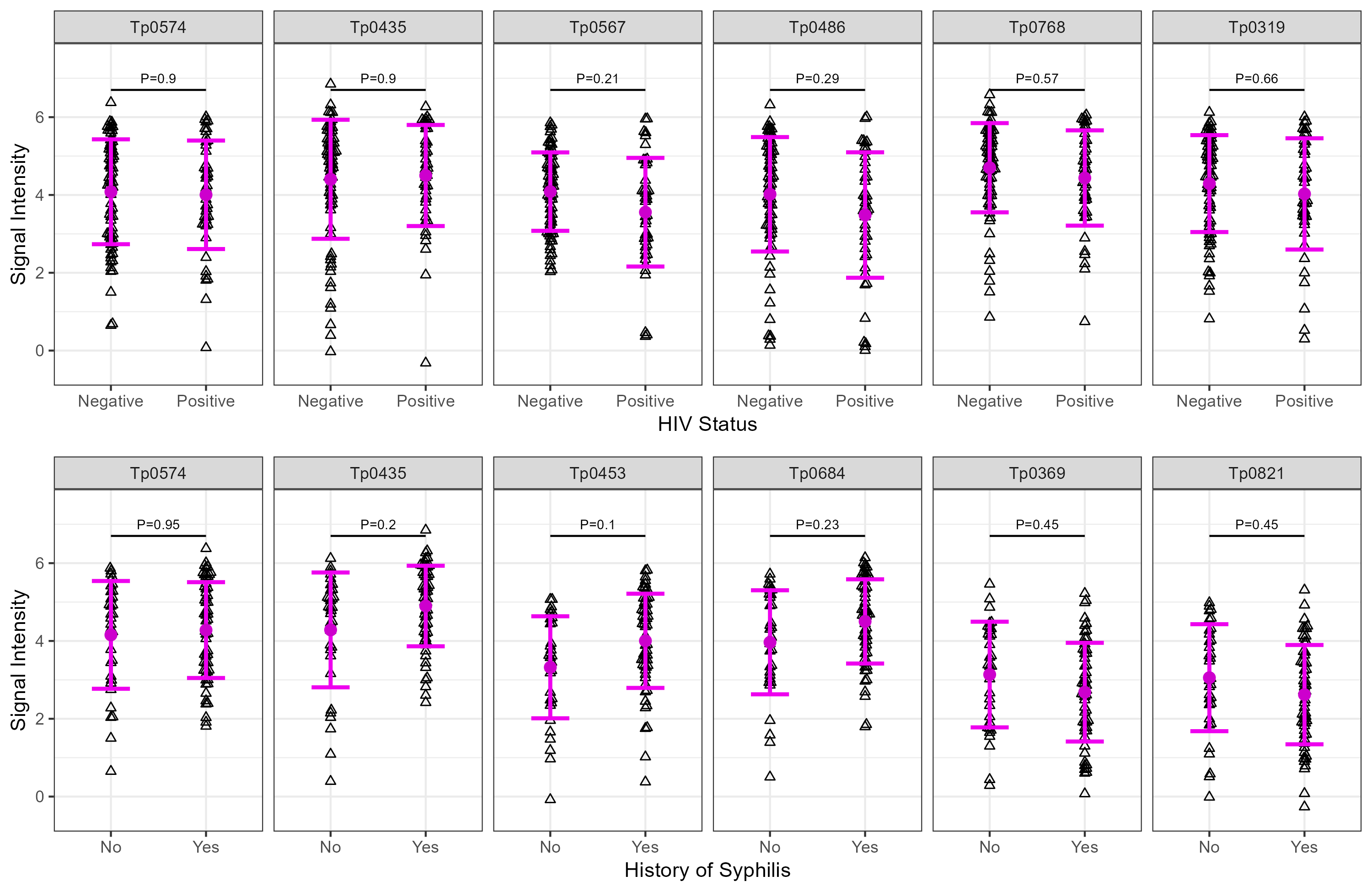

Supplement: Figure S3 — IgG reactivity to highly immunogenic T. pallidum proteins. [file mbio.00820-26-s0003.tif]

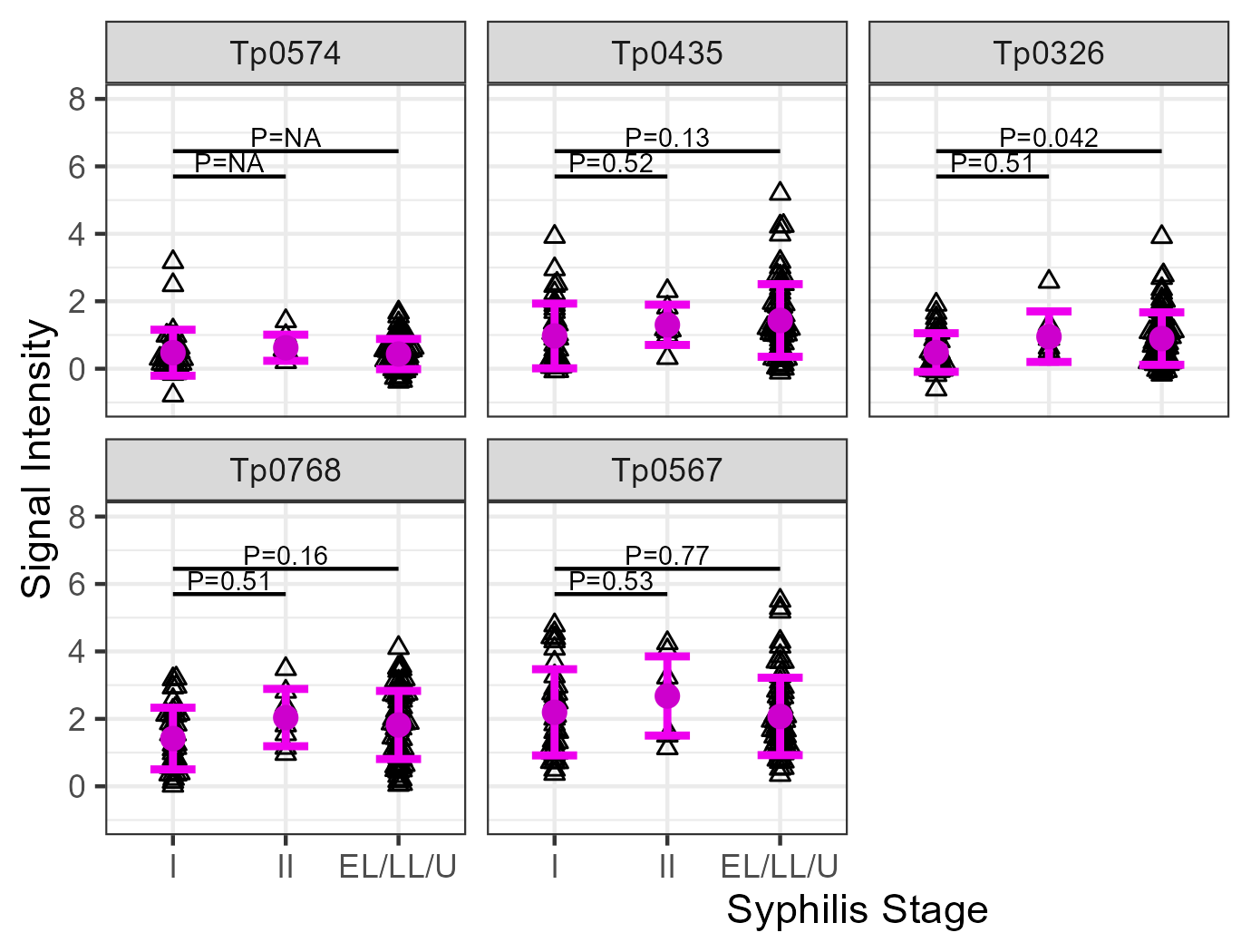

Supplement: Figure S4 — IgA reactivity to highly immunogenic T. pallidum antigens in pre-treatment sera. [file mbio.00820-26-s0004.tif]

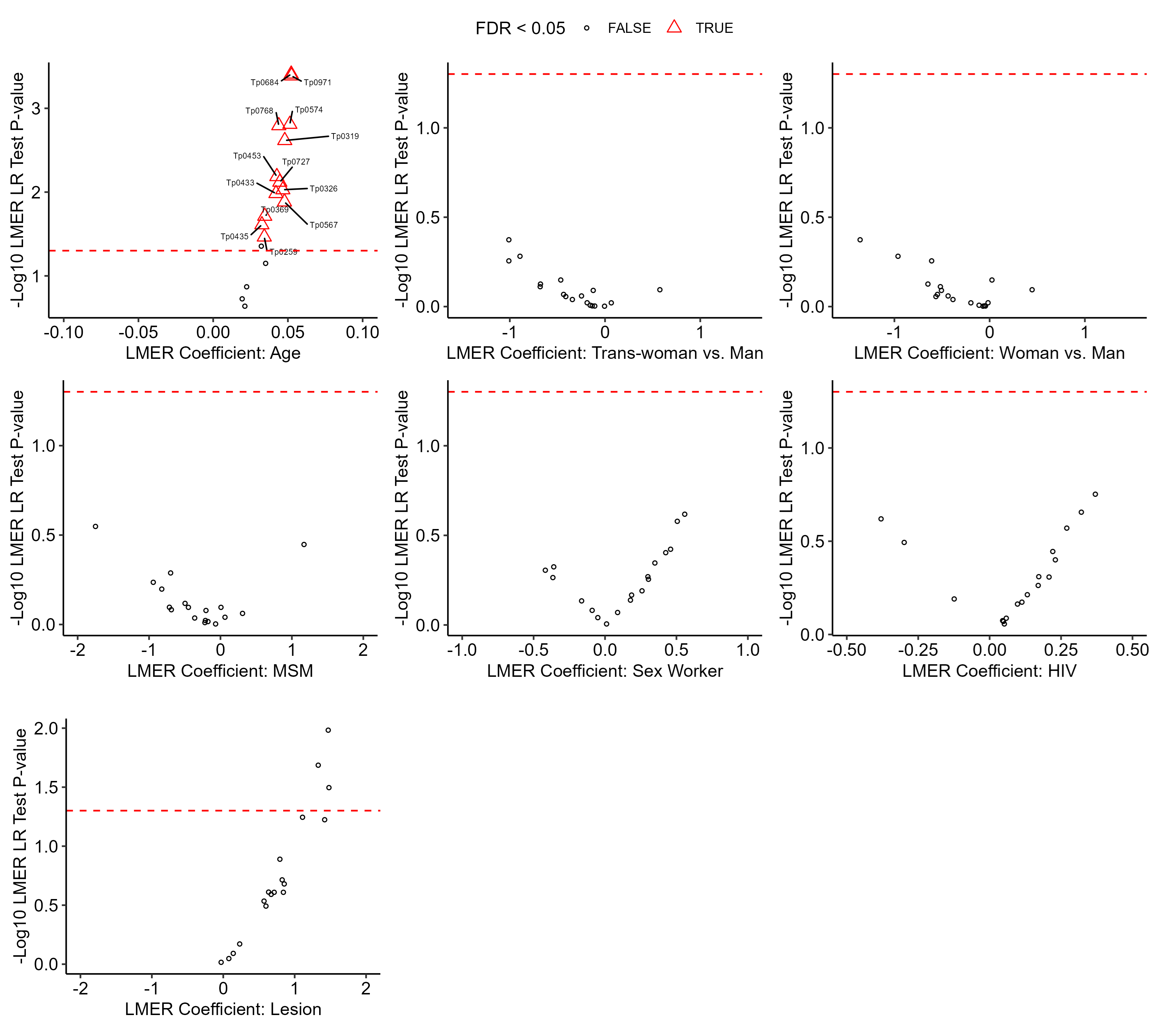

Supplement: Figure S5 — Effect of covariates on IgG antibody levels to selected T. pallidum antigens. [file mbio.00820-26-s0005.tif]

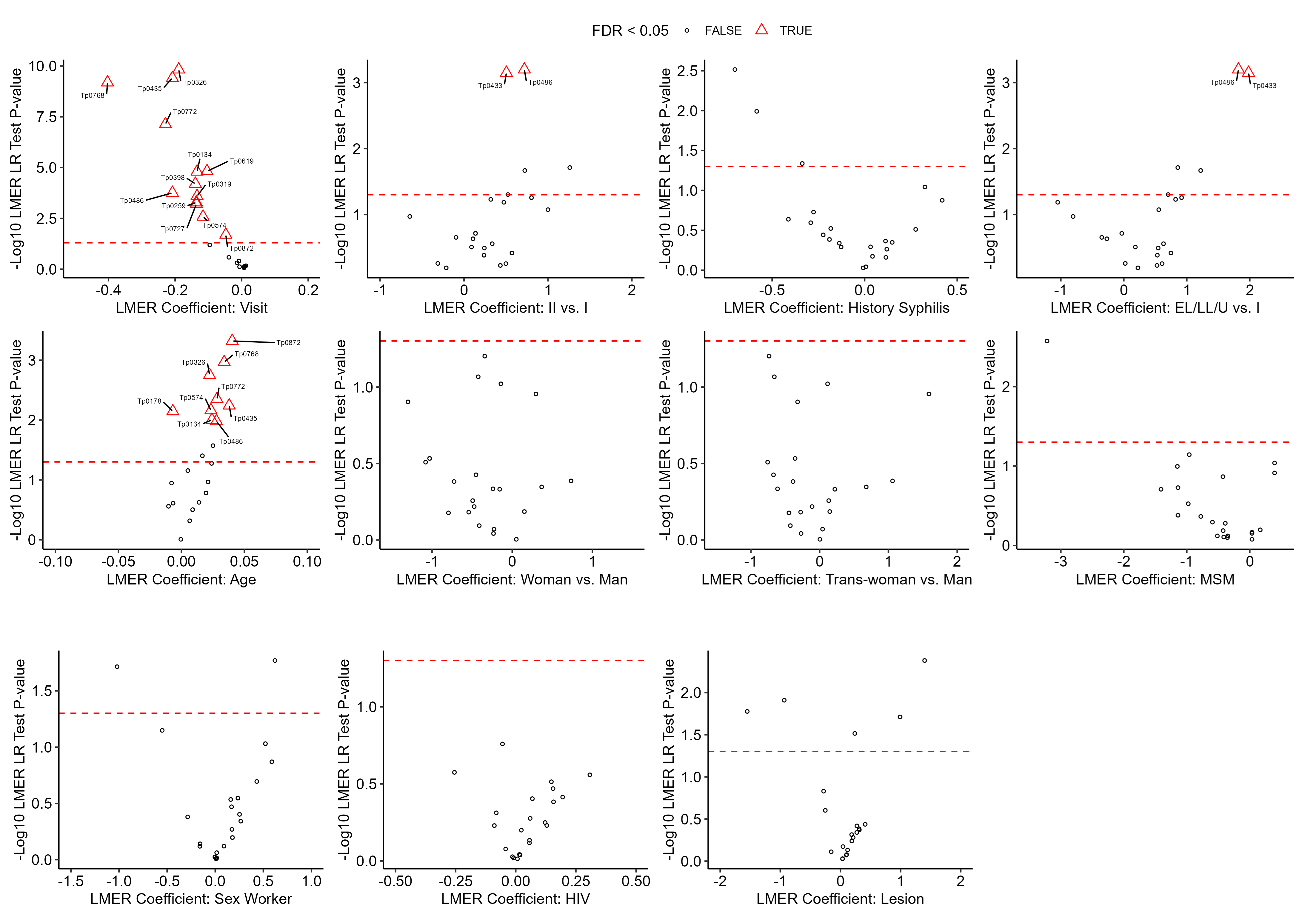

Supplement: Figure S6 — Effect of covariates on IgA antibody levels to selected T. pallidum antigens. [file mbio.00820-26-s0006.tif]
