# Supplementary material for: High-throughput profiling of the IgG and IgA response to the Treponema pallidum subsp. pallidum proteome in syphilis patients
Source: mBio. 2026 Jun 9;17(7):e00820-26. doi: 10.1128/mbio.00820-26 (PMC13343974; doi:10.1128/mbio.00820-26)
Supplement: Legends — for the supplemental figures. [file mbio.00820-26-s0007.docx]

**Figure S1. Overall IgG and IgA reactivity in control sera from non-syphilis patients and patients with periodontal disease.** (**A** and **B**) Interquartile range plots showing the maximal normalized IgG binding signal in non-syphilis sera (healthy controls) (**A**) and sera from periodontal disease patients (**B**) for each patient for each target on the array. Each bar represents a target on the array, and targets are ordered by the mean signal generated by testing each serum sample. Red bars represent printed proteins on the array with a significant seropositive response (i.e., above background) in at least 25% of the sera analyzed. Normalized IgG/IgA binding (Y axis) is expressed as the log2 signal-to-noise ratio, where a value of 0 represents specific antibody SI equal to the background, 1.0 represents twice the background, 2.0 represents 4-fold over background, and so forth. (**C** and **D**) Interquartile range plots showing the maximal normalized IgA binding signal in sera from non-syphilis patients (**C**) and periodontal disease patients (**D**).

**Figure S2. Random Forest analysis of protein features associated with antigen reactivity.** Random forest (RF) models were repeated 100 times randomly, resampling 90% of protein features. The percentage increase in mean square error (%incMSE) in 100 RF models is shown on the y-axis with a significance cutoff of 5% (horizontal red dashed line), and the percentage of times each antigen was identified as a significant variable is shown on the x-axis with a cutoff of 70% (vertical red dashed line). The protein features with mean %incMSI ≥5% and significance in ≥70% of RF models are labeled in the top right quadrant as significant associations with antigen reactivity.

**Figure S3: IgG reactivity to highly immunogenic *T. pallidum* proteins in pre-treatment sera by HIV status and history of syphilis.** The bee-swarm plots show IgG binding levels in HIV-negative and -positive patients and patients with or without history of syphilis in pre-treatment sera. Group means and standard error of mean (SEM) bars are shown in magenta. False discovery rate-adjusted P-values from univariate Student’s T tests between syphilis stage groups are shown above each comparison with the primary syphilis group.

**Figure S4: IgA reactivity to highly immunogenic *T. pallidum* antigens in pre-treatment sera by syphilis stage.** The bee-swarm plots show IgA binding levels in primary (N=38), secondary (N=8) and early latent, late latent or latent of unknown duration (N=70) patient sera before treatment. Group means and standard error of mean (SEM) bars are shown in magenta. False discovery rate-adjusted P-values from univariate Student’s T tests between syphilis stage groups are shown above each comparison with the primary syphilis group. Abbreviations: I, primary syphilis; II, secondary syphilis; EL/LL/U, early latent, late latent or latent syphilis of unknown duration.

**Figure S5. Effect of covariates on IgG antibody levels to selected *T. pallidum* antigens using linear mixed effects regression (LMER).** LMER models estimating the longitudinal trajectory of antibodies adjusted for age, gender, MSM, sex worker status, HIV, presence of lesions, history of syphilis and diagnosis of syphilis stage. The volcano plots show the LMER coefficient of the effect of covariates on antibody levels on the x-axis and inverse log_10_ P-value from likelihood ratio (LR) tests of LMER null vs full models on the y-axis. Responses associated with antibody levels after correction for the FDR are shown in red labeled triangles. The horizontal dashed line represents an unadjusted P-value of 0.05.

**Figure S6. Effect of covariates on IgA antibody levels to selected *T. pallidum* antigens using linear mixed effects regression (LMER).** LMER models estimating the longitudinal trajectory of antibodies adjusted for age, gender, MSM, sex worker status, HIV, presence of lesions, history of syphilis and diagnosis of syphilis stage. The volcano plots show the LMER coefficient of the effect of covariates on antibody levels on the x-axis and inverse log_10_ P-value from likelihood ratio (LR) tests of LMER null vs full models on the y-axis. Responses associated with antibody levels after correction for the FDR are shown in red labeled triangles. The horizontal dashed line represents an unadjusted P-value of 0.05.
